# Comparative Genomic Characterization of a Thailand–Myanmar Isolate, MS6, of *Vibrio cholerae* O1 El Tor, Which Is Phylogenetically Related to a “US Gulf Coast” Clone

**DOI:** 10.1371/journal.pone.0098120

**Published:** 2014-06-02

**Authors:** Kazuhisa Okada, Mathukorn Na-Ubol, Wirongrong Natakuathung, Amonrattana Roobthaisong, Fumito Maruyama, Ichiro Nakagawa, Siriporn Chantaroj, Shigeyuki Hamada

**Affiliations:** 1 Thailand–Japan Research Collaboration Center on Emerging and Re-emerging Infections (RCC-ERI), Nonthaburi, Thailand; 2 Research Institute for Microbial Diseases, Osaka University, Suita, Osaka, Japan; 3 Graduate School of Medical and Dental Sciences, Tokyo Medical and Dental University, Tokyo, Japan; 4 National Institute of Health, Department of Medical Sciences (DMSc), Ministry of Public Health, Nonthaburi, Thailand; Radboud University Medical Centre, NCMLS, Netherlands

## Abstract

**Background:**

The cholera outbreaks in Thailand during 2007–2010 were exclusively caused by the *Vibrio cholerae* O1 El Tor variant carrying the cholera toxin gene of the classical biotype. We previously isolated a *V. cholerae* O1 El Tor strain from a patient with diarrhea and designated it MS6. Multilocus sequence-typing analysis revealed that MS6 is most closely related to the U. S. Gulf Coast clone with the exception of two novel housekeeping genes.

**Methodology/Principal Findings:**

The nucleotide sequence of the genome of MS6 was determined and compared with those of 26 *V. cholerae* strains isolated from clinical and environmental sources worldwide. We show here that the MS6 isolate is distantly related to the ongoing seventh pandemic *V. cholerae* O1 El Tor strains. These strains differ with respect to polymorphisms in housekeeping genes, seventh pandemic group-specific markers, CTX phages, two genes encoding predicted transmembrane proteins, the presence of *metY* (MS6_A0927) or *hchA/luxR* in a highly conserved region of the *V. cholerae* O1 serogroup, and a superintegron (SI). We found that *V. cholerae* species carry either *hchA/luxR* or *metY* and that the *V. cholerae* O1 clade commonly possesses *hchA/luxR,* except for MS6 and U. S. Gulf Coast strains. These findings illuminate the evolutionary relationships among *V. cholerae* O1 strains. Moreover, the MS6 SI carries a quinolone-resistance gene cassette, which was closely related with those present in plasmid-borne integrons of other gram-negative bacteria.

**Conclusions/Significance:**

Phylogenetic analysis reveals that MS6 is most closely related to a U. S. Gulf Coast clone, indicating their divergence before that of the El Tor biotype strains from a common *V. cholerae* O1 ancestor. We propose that MS6 serves as an environmental aquatic reservoir of *V. cholerae* O1.

## Introduction

*Vibrio cholerae*, which is present in aquatic environments worldwide, is a facultatively anaerobic, asporogenous, motile, curved, or straight gram-negative rod. There are more than 200 serogroups of *V. cholerae*, but only serogroups O1 and O139 cause epidemics and pandemics of cholera in human populations [1], and cholera toxin causes the major clinical signs of the disease. The O1 serogroup is classified into classical or El Tor biotypes. The sixth cholera pandemic (1899–1923) was caused by the classical biotype, and the ongoing seventh cholera pandemic is caused by El Tor. Several other outbreaks of cholera occurred between the sixth and seventh pandemics, and some El Tor strains were isolated and are designated pre-seventh pandemic El Tor. During the past two decades, atypical *V. cholerae* O1 El Tor was isolated more frequently and was spread widely [2]–[6]. These isolates produce a cholera toxin that is distinct from that expressed by El Tor.

We isolated a strain from a clinical specimen that we designated MS6 that expresses the typical El Tor cholera toxin (genotype 3) [7]. Characterization of MS6 using ribotyping, pulsed-field gel electrophoresis, multiple-locus variable-number tandem-repeat analysis, and multilocus sequence typing analyses revealed that the strain is not closely related to other strains isolated in Thailand or other countries. The sequences of MS6 housekeeping genes reveal that it is most closely related to *V. cholerae* O1 strains isolated in the U. S. Gulf Coast area. The U. S. Gulf Coast clone [8], [9] is genetically distinct from several pathogenic clones of *V. cholerae* O1 [10], which caused only sporadic disease or small outbreaks, with no transmission spread along the Gulf Coast [11]. Nevertheless, two of 15 housekeeping genes of MS6 (*malP* and *pepN*), are minimally related to those of U. S. Gulf Coast strains and represent novel sequences according to nucleotide sequence comparisons using BLAST.

Here, we report the characterization of entire genome of *V. cholerae* O1 El Tor strain MS6. The results of these analyses enhance our understanding of the evolution and genetic basis of the pathogenicity of *V. cholerae*.

## Materials and Methods

### Ethics Statement

The patient’s consent was not required by the hospital, because the isolation of *V. cholerae* was performed as part of clinical management during hospitalization. To protect the privacy of the patient and the patient’s family, all identifying information was excluded from this study.

### Strains, Growth Conditions, and DNA Isolation

*V. cholerae* O1 El Tor serotype Ogawa strain MS6 was isolated from a Myanmanese inpatient suffering from diarrhea who was treated at a hospital located in a Thai–Myanmar border city [12]. MS6 was grown in Tryptic Soy Broth (Difco, Detroit, MI) at 37°C for 18 h with shaking. Cells were collected by centrifugation, and genomic DNA was extracted using proteinase K and phenol/chloroform, treated with RNase, and purified.

### Genome Sequencing, Assembly, and Annotation

The genome of MS6 was sequenced using the Roche GS FLX Titanium system (8-kb-span paired-end library). Newbler (version 2.6; 454 Life Sciences/Roche, Branford, CT) was used to generate and assemble 395,285 reads into two scaffolds (2.95 Mb and 1.11 Mb) comprising 66 contigs and 53 stand-alone contigs ≥500 bp with an average read depth of 24.5. The gaps between contigs were closed using the unassembled mate-paired reads, PCR sequencing, or both of amplicons generated using primers flanking the gaps. Further, Illumina sequence data (14.5 Gbases, 100-bp paired-end reads) was used to improve low quality regions using GenomeTraveler (In Silico Biology, Inc., Yokohama, Japan). The whole genome sequence of MS6 was deposited in the DDBJ (AP014524/AP014525).

The RAST server (ver. 4.0) [13] automatically generated annotations of the 26 reference and MS6 genomes. Accession numbers for complete and draft genome sequences are as follows: CP001233.1/CP001234.1, AE003852.1/AE003853.1, CP003069.1/CP003070.1, ACHX00000000, NC_012667/NC_012668, ACHZ00000000, ACVW00000000, AAKF03000000, AAUT01000000, ACIA00000000, ACFQ00000000, ACHW00000000, AAUS02000000, AAWD01000000, CP000626/CP000627, ADAI00000000, AAUR00000000, AAWF01000000, AAKI02000000, AAKJ02000000, AAUU01000000, AATY01000000, ACHV00000000, ACHY00000000, AAKH03000000, AAWG00000000. Annotations were compared using the SEED viewer (ver. 4.0) [14] and in silico Molecular Cloning Genomics Edition (IMC-GE, ver. 5.2.6D; In Silico Biology, Inc.). Using the Clusters of Orthologous Groups (COG) database [15], we assigned functions to each protein family encoded by the MS6 genome. Annotation of the MS6 genome encoding CTX phage sequences and *qnr* were manually curated to incorporate evidence from published articles and public databases.

### Phylogenetic Analyses

Coding sequences (CDSs) present as a single copy in 27 genomes were analyzed using the pan-genomes analysis pipeline (PGAP) 1.02 [16] with the default parameters. CDSs of the same length (including gaps) were aligned after using a MAFFT with L-INS-I strategy [17]. Further, we chose CDSs with a low probability of recombination based on the PHI-test (cutoff value: p-value ≥0.05) in SplitsTree4 [18]. Subsets of predicted amino acid sequences of each strain were concatenated, and maximum likelihood analyses were conducted with 100 bootstrap replicates by using the Randomized Axelerated Maximum Likelihood (RAxML) program [19]. The results were visualized using Dendroscope 3 [20].

### Design of Primers and Conditions for Detection of the *V. cholerae* Molecular Markers *metY* and *hchA/luxR*

PCR primers were designed to amplify *metY* or *hchA/luxR* by using the sequences of the 27 *V. cholerae* genomes. The PCR reactions employed two forward primers (*metY*-F, 5′-GCGTGAAACCGGAGATGATCC-3′ and *luxR*-F, 5′-TAGCTCACCGCGAGCTCGTTG-3′) and one reverse primer (*lys-R,* 5′*-*AGCGCAGAAGGTGTTACGCCA-3′). The theoretical amplicon lengths for *metY* and *hchA/luxR* are 353 bp and 521 bp, respectively. All amplification mixtures consisted of template DNA, 0.2 µM of each primer, 200 µM of each deoxynucleoside triphosphate, and 0.025 U/µl of Ex Taq polymerase in the buffer supplied with the enzyme. After an initial denaturation step of 94°C for 5 min, the reaction was conducted using 30 cycles each of 94°C for 30 s, 59°C for 30 s, and 72°C for 30 s. PCR products were analyzed by electrophoresing the products on 1.5% agarose gels, and the amplicons were detected using ethidium bromide.

## Results and Discussion

### Comparison of the Genomes of *V. cholerae* O1 El Tor MS6 and Reference Strains

The *V. cholerae* MS6 genome consists of circular chromosomes 1 and 2, comprising 2,936,971 bp and 1,093,973 bp with average G+C contents of 47.7% and 46.8%, respectively. RAST annotation analysis of the MS6 genome predicted 3,746 predicted open reading frames (ORFs). The nucleotide sequences of the genes encoding the components of the polysaccharide (*wav* cluster) and O antigen (*wbe* gene cluster) biosynthetic pathways [21] were highly similar to those of other O1 El Tor strains, indicating that other organisms were not likely their source.

**Figure 1:**
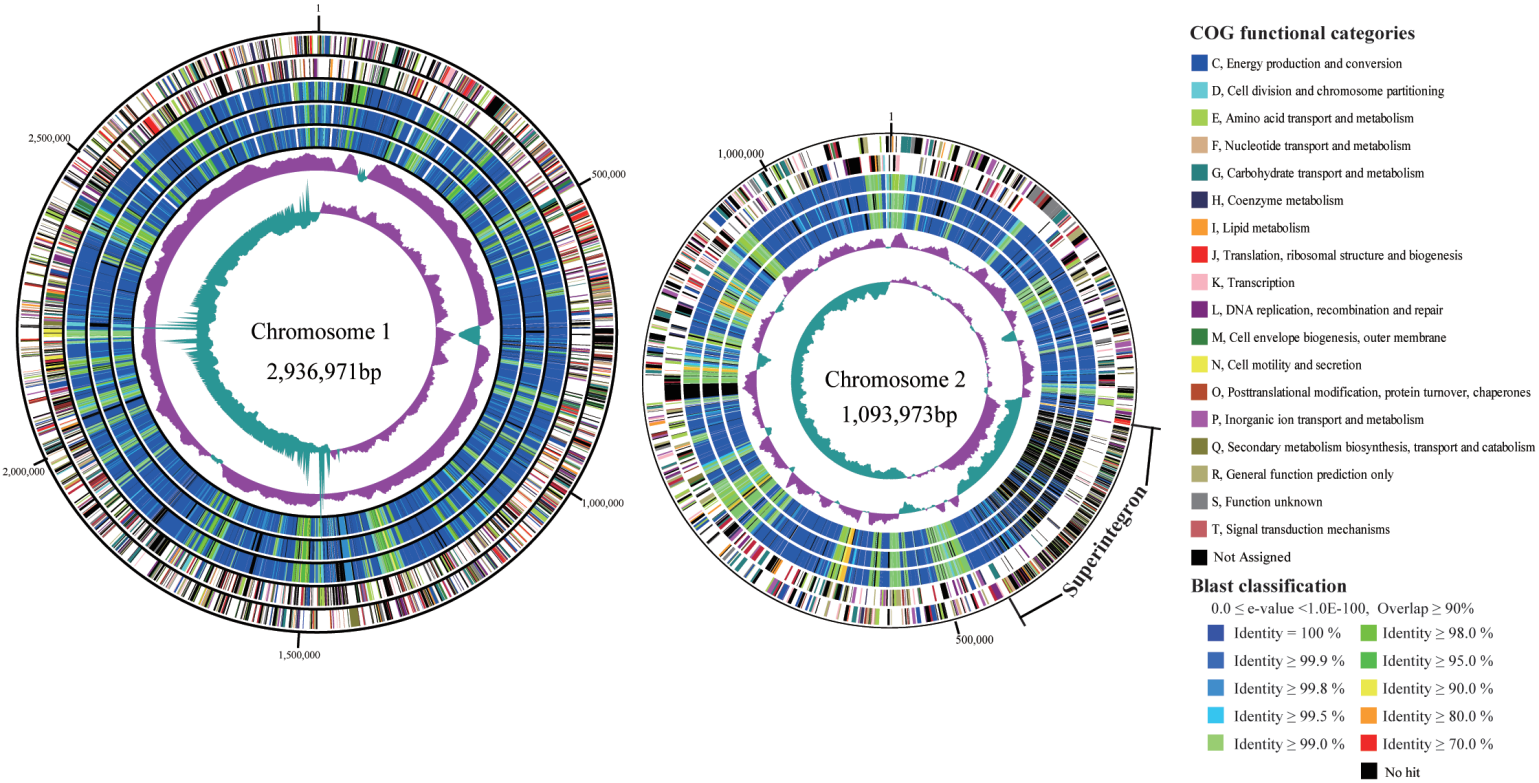
Comparison of the large and small chromosomes of *Vibrio cholerae* O1 El Tor MS6 and those of three reference strains. The first and second outermost circles of each chromosome show the COG functional categories of the MS6 coding regions, in the clockwise and anticlockwise directions, respectively. The next three circles compare the coding regions of *V. cholerae* O1 M66-2, N16961, and 2010EL-1786 with those of MS6. The sixth and seventh circles show the GC content of the MS6 sequence and the percent G+C deviation by strand, respectively.

We next compared the genome sequences of MS6 with those of the prototype seventh pandemic El Tor, N16961 [22], seventh pandemic atypical El Tor, 2010EL-1786 [23], and the pre-seventh pandemic El Tor, M66-2 [24] strains deposited in the EMBL/GenBank/DDBJ databases. We identified 3,420 core ORFs based on comprehensive orthologous gene detection using reciprocal comparison. ORFs of chromosome 1 were shared more frequently with those of the four test strains compared with chromosome 2. The sequence of the superintegron (SI), which functions as a gene capture system, varied considerably among the strains (Fig. 1). The gene order of the common ORFs of each chromosome (i.e., synteny) was well conserved, except for a 184-kb inversion near the replication origin of chromosome 1 in strains MS6 and 2010EL-1786 compared with the other two strains.

The sequences of the chromosomes 1 and 2 ORFs were compared with the genomes of three *V. cholerae* O1 reference strains. The shared ORFs were classified according to the percentage identity of the DNA sequences (Fig. 1, outer rings 3–5). Blocks of ORFs (green represents 95% and 99% identities) were recognized in the chromosomes of each strain. Further, some of the ORFs were highly conserved only in the pre-seventh pandemic strain M66-2 or seventh pandemic strains N16961 and 2010EL-1786. Therefore, the MS6 genome exhibits a mosaic structure, which was likely generated by homologous recombination with other *V. cholerae* chromosomes. Sixteen ORFs and 44 ORFs on the large and small chromosomes, respectively, of MS6 were not detected in the genomes of the three reference strains. Notably, 51 of these 60 ORFs are encoded by mobile genetic elements or the SI.

The MS6 ORFs included in the 18 COG categories were compared with the complete genome sequences of the three *V. cholerae* O1 strains. The number of ORFs in these three strains that were identical to those of MS6 was examined in proportion to the total number of ORFs in each category (results not shown). The average percentage of amino acid and nucleotide sequence matches among chromosome 1 of all categories were 79% and 69%, respectively; however, similarities of ORFs on chromosome 2 were approximately 10% lower than those of chromosome 1. We compared the COG-categorized ORFs of MS6 with those of 26 strains of *V. cholerae* (Fig. S1). Only the ORFs of *V. cholerae* O1 were highly related to those of MS6.

**Figure 2:**
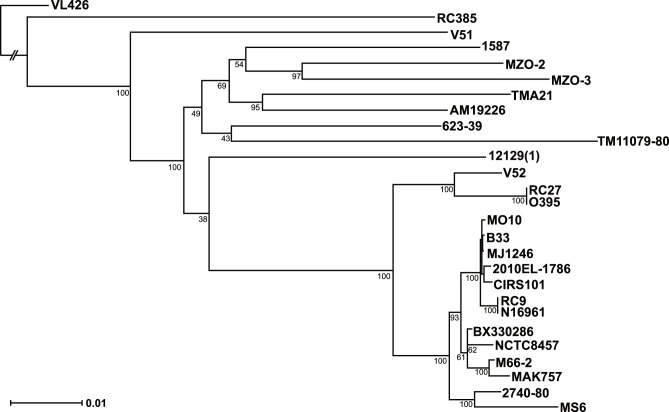
Maximum-likelihood tree showing the phylogenetic relationships among MS6 and 26 *Vibrio cholerae* strains representing diverse serogroups. The tree was rooted by treating the VL426 strain as an outlier. Bootstrap supports (%) are indicated at the branching points. Branch lengths are proportional to the sequence differences.

The relationships among the 27 strains were further investigated using genome-wide phylogenetic analysis (Fig. 2). All *V. cholerae* O1 strains except two comprised a clade (PG clade), and 16 strains of the PG clade were further divided into two subclades [25]. The PG-1 subclade comprises most of the *V. cholerae* O1 El Tor strains and MO10 (O139), whereas the PG-2 subclade includes two classical strains and VC52 (O37). MS6 is most closely related to the U. S. Gulf Coast strain 2740-80, indicating that these organisms diverged before the phylogenetic separation of the El Tor biotype strains from a common *V. cholerae* O1 ancestor.

### Significant Features of the MS6 Genome

Two tandem copies of CTX prophages are present at the dimer resolution site (*dif*) of MS6 chromosome 1 (Fig. S2). The toxin-linked cryptic element is present within the *dif1* region of MS6, indicating that these elements likely integrated into the host chromosome through XerC/XerD-mediated recombination [26]–[28]. No CTX prophage was detected at MS6 *dif2*. The 6.9-kb CTXφ genome includes a DNA replication module designated repeat sequence (RS) 2, which comprises *rstR, rstA*, *rstB* and a core region comprising *psh, cep, gIII (orfU), ace, zot*, and *ctxAB* [29]. The sequences of these MS6 and O1 El Tor strain N16961 genes are identical.

However, the intergenic region-1 (ig-1) located near *rstR* and the toxboxes required for activating transcription from the cholera toxin promoter (P*ctxAB*) [30], [31] differed between these strains. Specifically, MS6 possesses three perfect direct repeats (TTTTGAT) within P*ctxAB* as well as strains MAK757, MO10, V52, RC9, and CIRS101, and strain N16961 harbors four. The ig-1 region in MS6 is longer than that of N16961. Annotation using IMC-GE predicted that each ig-1 region of the CTX prophage encodes a protein (CTXUG-1) composed of 91 amino acid residues. Further, although MS6 lacks an RS1 region (consisting of *rstR, rstA, rstB*, and *rstC*), which is usually associated with CTXφ in *V. cholerae* O1 El Tor and O139 isolates [32], MS6 possesses a genomic island designated MS6CTXAGI that encodes a similar *rstC* sequence (two amino acid residue differences compared with N16961), four ORFs of unknown function, a putative transcriptional regulator, and a CTXUG-1 homologue.

**Figure 3:**
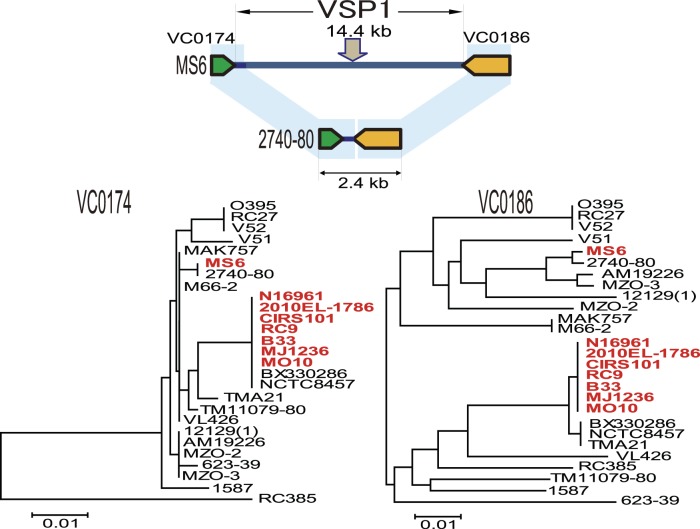
MS6, but not U. S. Gulf Coast strain 2740-80, carries the *Vibrio* seventh pandemic island-1 (VSP-1) between *VC0174* and *VC0186*. Dendrograms were constructed based on the genes flanking VSP-1 (*VC0174* and *VC0186*) using the neighbor-joining method using MEGA version 5.2 [37]. VSP-1 was identified in the eight *V. cholerae* strains shown in red. MS6 as well as the seventh pandemic strains carry the full VSP-1 sequence between *VC0174* and *VC0186*, which is closely related to that of strain 2740-80. Scale bars indicate nucleotide substitutions per site.

Toxin coregulated pilus, which acts as a receptor for phage CTX, is essential for colonizing infant mice as well as humans in model systems and is encoded by sequences within a 45-kb pathogenicity island (VPI-1) [33]. The *Vibrio* pathogenicity island-2 (VPI-2; VC1758–VC1809; 57.3 kb) [34] that encodes neuraminidase and proteins involved in sialic acid metabolism is present in MS6. VPI-1 and VPI-2 regions in MS6 are highly related to those of the other *V. cholerae* O1 strains. However, the *Vibrio* seventh pandemic island-1 (VSP-1; VC0175–0185; 14 kb) [32], [35], [36] and *Vibrio* seventh pandemic island-2 (VSP-2; VC0490–VC0516; 27 kb) differ between MS6 and seventh pandemic strains. The MS6 genome lacks VSP-2, and although the entire VSP-1 region is present, its flanking genes *VC0174* and *VC0186* are distantly related to those of seventh pandemic strains (Fig. 3). The dendrograms based on their sequences indicate that they were closely related to those of 2740-80.

We identified a novel 4.7-kb mobile genetic element designated M1 in MS6, which is integrated into the spacer region between *rpmF* and *maf* on the large chromosome (Fig. S3). MS6-M1 comprises six ORFs (MS6_1784 to MS6_1789), including a putative integrase. The outer membrane protein of MS6 is encoded by *ompW*, which is split by the transposon (Fig. S4). In contrast, 11 bp of *ompW* is deleted in strain 2740-80. This gene is conserved among *V. cholerae* strains and is utilized for identification of *V. cholerae* strains [38]. Although the biological role of *ompW* is unknown, its function may be linked to the adaptive response to stress [39]. Among the 27 strains studied here, the integral membrane protein MS6_A0359 with four transmembrane-spanning helices (motif HPP) [40] is present in strains MS6, 2740-80, and R385. Moreover, the putative RNA-binding protein MS6_A0295 is present only in strains MS6 and 2740-80 in the PG clade, whereas an MS6_A0295 homologue is present in 10 of 11 strains in distinct phyletic lineages.

### The SI of MS6 is a Massive Gene Capture System

**Figure 4:**
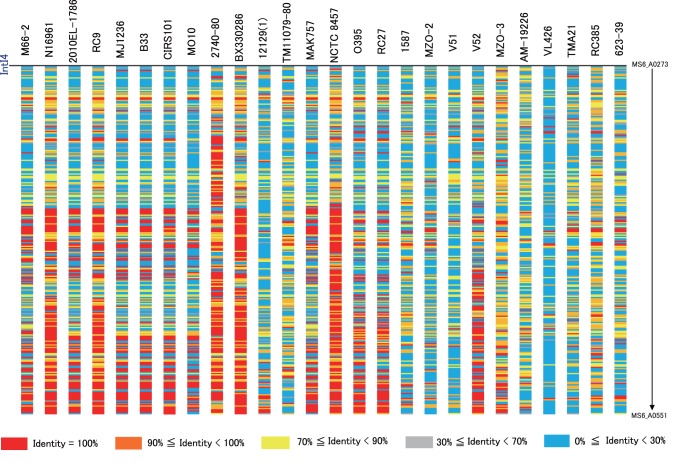
Distribution of open reading frames (ORFs) of the MS6 SI among the 26 reference strains. The 279 ORFs (MS6_A0273 to MS6_A0551) in the 144-kb SI of MS6 were compared with the genomes of 26 *V. cholerae* strains and were classified based on the percentage amino acid sequence identity, and colored accordingly. The ORFs of MS6 are highly similar to those of the U. S. Gulf Coast strain 2740-80.

The SI of *V. cholerae* O1 strain N16961 is a 127-kb integron island that resides on the small chromosome (VCA0291–VCA0508) [22]. Most genes predicted to reside within the SI encode hypothetical proteins, and the SI may serve as a source of genetic variation. Strains 2010EL-1786 and M66-2 harbor approximately 100-kb SI regions compared with the 144-kb SI of MS6. The RAST server automatically annotated 46 ORFs in category R of the SI that represent the death-on-curing family of toxin proteins, which contain the well-conserved central motif HxFx[ND][AG]NKR [41]. We detected 39, four, and two ORFs of this family, respectively, in N16961, M66-2, and 2010EL-1786, and we therefore suggest that toxin–antitoxin modules plays a role in maintaining the large SI in MS6. All predicted ORFs of the SI of MS6 were compared with those of 26 reference *V. cholerae* genomes (Fig. 4). ORFs identical or similar to those of MS6 were present in the genomes of strains 2740-80, BX330286, and NCTC8457. In contrast, there was little similarity between the MS6 ORFs and those of most of the non-O1/non-O139 strains. The similar organization of ORFs within their SI domains suggests a close relationship between MS6 and strain 2740-80.

### A *qnr* Cassette is Present in the SI of MS6

On the small chromsome of MS6, *qnr* is located approximately 28 kb from the SI integrase (IntI4). The *qnr* cassette was not detected in the chromosomes of other *V. cholerae* strains [42]. The MS6 *qnr* nucleotide sequence is 99% (650/657) identical to that of *qnrVC4*, which is a novel complex class 1 integron harboring the ISCR1 element in an aquatic isolate of *Aeromonas punctata* [43]. Moreover, the gene cassette in the class 1 integron harbored *attC* and a 214-bp noncoding sequence, which were a nearly perfect match to the gene cassette of MS6 (Fig. S5). This finding provides evidence that this gene cassette was mobilized from the SI into a plasmid-borne integron through class 1 integrase activity [44], leading to the transmission of the resistance integron to several gram-negative bacteria.

### *MetY* is Present in MS6 and U. S. Gulf Coast Strains

**Figure 5:**
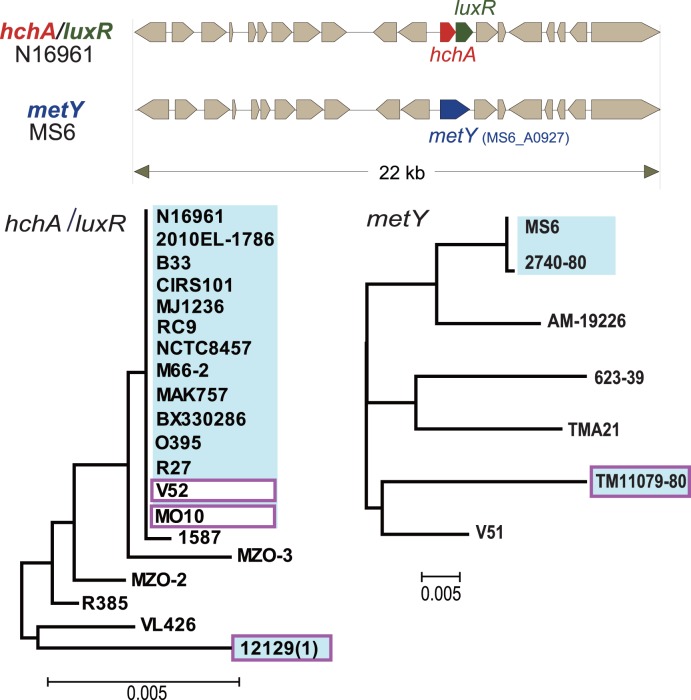
*Vibrio cholerae* O1 genomes can be divided into two clusters that possess either *hchA/luxR* or *metY* in a conserved syntenic region of the small chromosome. Dendrograms were constructed based on *hchA*/*luxR* or *metY* using the method described in the legend to Fig. 3. Strains highlighted in blue belong to serogroup O1. However, four strains enclosed in the purple square may have undergone lateral gene exchange of O-antigen gene clusters; thus, strains V52 and MO10 were converted into O37 and O139 serogroups, respectively, while strains 12129(1) and TM11079-80 gained the O1-antigen gene cluster [25].

All *V. cholerae* strains carry either *hchA/luxR* or *metY* between MS6_A0926 and MS6_A0928 on the small chromosome (Fig. 5). The PG clade, except for strains 2740-80 and MS6, harbors *hchA/luxR*, and *metY* is present only in MS6 and strain 2740-80. Although strains VL426, MZO-2, MZO-3, R385, 1587, and 12129(1) belong to distinct phyletic lineages, they harbor *hchA*/*luxR*. Moreover, the synteny of the surrounding regions, DNA sequences, or both are distinguishable from those of the O1 lineage.

**Figure 6:**
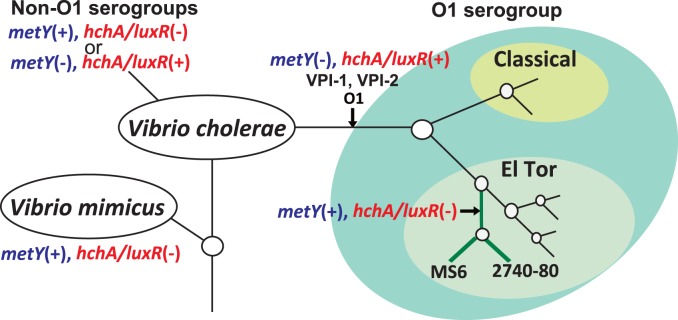
Hypothetical evolutionary relationship among clades of *Vibrio cholerae* with reference to strain MS6. Hypothetical ancestral *Vibrio* organisms are indicated by open circles. Although *V. cholerae* O1 possesses the partially overlapping *hchA/luxR*, they are replaced by *metY* in strains MS6 and 2740-80.

The 122 isolates of *V. cholerae* O1 from a variety of sources harbor *hchA/luxR* but not *metY*. In contrast, *hchA/luxR* is present in 47 of 64 non-O1/non-O139 *V. cholerae* isolates from clinical and environmental sources, and *metY* is present in the remaining 17 strains. Further, the sequences of *hchA/luxR* of 40 selected isolates of *V. cholerae* O1 El Tor are identical, including *V. cholerae* O1 El Tor strains N16961 and MAK757 (Fig. 5). These findings suggest that strain 2740-80 and MS6 reverted to *metY* from *hchA/luxR* in the distant past (Fig. S6, Fig. 6). Sequence comparisons of stains of the closely related species *V. mimicus* detected *metY* but not *hchA/luxR* in the genomes of strains VM573, SX-4, VM603, MB-451, and VM223.

The amino acid sequence of MS6_A0927 was 52% identical (68% similar) to the product (O-acetyl-homoserine sulfhydrylase; EC 2.5.1.49) of *metY* in *Leptospira meyeri* [46]. The predicted amino acid sequence of *Escherichia coli hchA*, which encodes heat shock protein 31 (Hsp31) [47], [48], is 60% identical (similarity, 78%) to that of *V. cholerae* O1. Moreover, evidence indicates that Hsp31 contributes to the resistance to acid of stationary-phase *E. coli* [49]. Acid tolerance represents a significant factor in the epidemic spread and virulence of *V. cholerae* [50].

## Conclusions

The analysis of the complete genome of MS6, which is distantly related to pathogenic O1 El Tor strains of *V. cholerae*, contributes insights into the evolution of the *V. cholerae* O1 serogroup as well as others. Our approaches demonstrates that chromosomes 1 and 2 of MS6 were frequently modified by horizontal gene transfer from other *Vibrio* species after divergence from a common ancestor of the PG-1 subclade and MS6. The genomic features of MS6 are most similar to those of U. S. Gulf Coast strain 2740-80.

## Supporting Information

Figure S1 **Clusters of orthologous group classes of the open reading frames (ORFs) of** ***V. cholerae*** **MS6 are similar to those of** ***V. cholerae*** **O1 strains but not to those of the non-O1 serogroup.** The 2,548 ORFs of the MS6 genome were assigned to 18 COG functional categories, and the numbers of ORFs are shown in parentheses in the right column. The percentage identities among the ORFs were calculated by dividing the number of identical ORFs of each reference strain genome by the number of MS6 ORFs of each functional category. Strains highlighted in blue and red belong to serogroups O1 and non-O1, respectively. We suggest that the four strains enclosed in the purple square underwent lateral gene exchange of O-antigen gene clusters (Fig. 5).
(EPS)

Figure S2 **The MS6 CTX prophage and adjacent genetic elements.** The MS6 genome harbors two CTX prophage sequences between MS6_1229 and MS6_1252. The genomic island MS6CTXAGI (CTX phage-Associated Genomic Island), which comprises seven open reading frames (ORFs) as well as one encoding an RstC homolog, resides between *rtxA* and the CTX prophage. The lower panel shows the amino acid sequences of the three genes encoding CTXUG-1.
(EPS)

Figure S3 **Genome structure of the mobile genetic element MS6-M1.** MS6-M1 resides in the spacer region between *rpmF* (green) and *maf* (blue) on the large chromosome (ChI) and harbors 5 ORFs (orange) and a putative integrase (red). The direct repeats (arrowheads) are separated by 4.7 kb. MS6-M1-like structures were detected only in the superintegron (SI) region of the classical stains O395 and R27, although these two regions are interrupted by the transposon *orfAB*. The percentage nucleotide sequence identities among the integrase and the four ORFs are 73.2% and 89.5%, respectively.
(EPS)

Figure S4 **A transposable element disrupts MS6** ***ompW.*** An outer membrane protein of strain N16961 is encoded by *ompW* (VCA0867, 654 bp). The homologous gene of MS6 is interrupted by an insertion of 1.2 kb encoding a transposase, and 11 bp were deleted from the corresponding allele of strain 2740-80.
(EPS)

Figure S5 **The superintegron (SI) of MS6 harbors a quinolone-resistance gene cassette (** ***qnrVC4*** **) present in the class 1 integron of** ***Aeromonas punctata*** **159.** The *qnrVC4* cassette of MS6 is located between *V. cholerae* repeats (VCRs are indicated in italics in the DNA sequence). VCR represents the attachment C site (*attC*) associated with the captured cassette in the SI of *V. cholerae*. VCR and a noncoding region corresponding to the gene cassette of the class 1 integron are linked to *qnrVC4.* The sequences of the latter two elements are 97% identical (988/1021).
(EPS)

Figure S6 **Evidence for the substitution of** ***hchA/luxR*** **or** ***metY*** **in highly conserved regions.** The nucleotide sequences of an approximately 22-kb region containing *hchA/luxR* or *metY* in MS6, 2740-80 (U. S. Gulf Coast), M66-2 (pre-seventh pandemic), and O395 (classical) strains were aligned using BioEdit version 7.1.3.0 [45]. The location of *metY* and *hchA/luxR* are highlighted in blue and red, respectively. Identical nucleotides are indicated by dots. The distribution of sequence differences (mismatches and gaps (−) is most frequent near *hchA/luxR* and *metY*, whereas the regions upstream and downstream of these genes are highly conserved.
(PDF)

## References

[1] Sack DA, Sack RB, Nair GB, Siddique AK (2004) Cholera. Lancet 363: 223–233. 14738797 10.1016/s0140-6736(03)15328-7

[2] Nair GB, Qadri F, Holmgren J, Svennerholm AM, Safa A, et al (2006) Cholera due to altered El Tor strains of *Vibrio cholerae* O1 in Bangladesh. J Clin Microbiol 44: 4211–4213. 16957040 10.1128/JCM.01304-06 PMC1698305

[3] Safa A, Sultana J, Dac Cam P, Mwansa JC, Kong RY (2008) *Vibrio cholerae* O1 hybrid El Tor strains, Asia and Africa. Emerg Infect Dis 14: 987–988. 18507925 10.3201/eid1406.080129 PMC2600311

[4] Safa A, Nair GB, Kong RY (2010) Evolution of new variants of *Vibrio cholerae* O1. Trends Microbiol 18: 46–54. 19942436 10.1016/j.tim.2009.10.003

[5] Okada K, Chantaroj S, Roobthaisong A, Hamada S, Sawanpanyalert P (2010) A cholera outbreak of the *Vibrio cholerae* O1 El Tor variant carrying classical CtxB in northeastern Thailand in 2007. Am J Trop Med Hyg 82: 875–878. 20439970 10.4269/ajtmh.2010.09-0537 PMC2861402

[6] Na-Ubol M, Srimanote P, Chongsa-Nguan M, Indrawattana N, Sookrung N, et al (2011) Hybrid & El Tor variant biotypes of *Vibrio cholerae* O1 in Thailand. Indian J Med Res 133: 387–394. 21537091 PMC3103171

[7] Okada K, Roobthaisong A, Nakagawa I, Hamada S, Chantaroj S (2012) Genotypic and PFGE/MLVA analyses of *Vibrio cholerae* O1: geographical spread and temporal changes during the 2007–2010 cholera outbreaks in Thailand. PLoS One 7: e30863. 22292065 10.1371/journal.pone.0030863 PMC3265523

[8] Kaper JB, Bradford HB, Roberts NC, Falkow S (1982) Molecular epidemiology of *Vibrio cholerae* in the U.S. Gulf Coast. J Clin Microbiol 16: 129–134. 7107852 10.1128/jcm.16.1.129-134.1982 PMC272308

[9] DePaola A, Capers GM, Motes ML, Olsvik O, Fields PI, et al (1992) Isolation of Latin American epidemic strain of *Vibrio cholerae* O1 from U. S. Gulf Coast. Lancet 339: 624. 10.1016/0140-6736(92)90917-r 1347133

[10] Salim A, Lan R, Reeves PR (2005) *Vibrio cholerae* pathogenic clones. Emerg Infect Dis 11: 1758–1760. 16318732 10.3201/eid1111.041170 PMC3367346

[11] Wachsmuth IK, Bopp CA, Fields PI, Carrillo C (1991) Difference between toxigenic *Vibrio cholerae* O1 from South America and US gulf coast. Lancet 337: 1097–1098. 1673518 10.1016/0140-6736(91)91744-f

[12] Okada K, Roobthaisong A, Swaddiwudhipong W, Hamada S, Chantaroj S (2013) *Vibrio cholerae* O1 with a novel genetic background, Thailand-Myanmar. Emerg Infect Dis 19: 1015–1017. 23735934 10.3201/eid1906.120345 PMC3713811

[13] Aziz RK, Bartels D, Best AA, DeJongh M, Disz T, et al (2008) The RAST Server: rapid annotations using subsystems technology. BMC Genomics 9: 75. 18261238 10.1186/1471-2164-9-75 PMC2265698

[14] Overbeek R, Begley T, Butler RM, Choudhuri JV, Chuang HY, et al (2005) The subsystems approach to genome annotation and its use in the project to annotate 1000 genomes. Nucleic Acids Res 33: 5691–5702. 16214803 10.1093/nar/gki866 PMC1251668

[15] Tatusov RL, Galperin MY, Natale DA, Koonin EV (2000) The COG database: a tool for genome-scale analysis of protein functions and evolution. Nucleic Acids Res 28: 33–36. 10592175 10.1093/nar/28.1.33 PMC102395

[16] Zhao Y, Wu J, Yang J, Sun S, Xiao J, et al (2012) PGAP: pan-genomes analysis pipeline. Bioinformatics 28: 416–418. 22130594 10.1093/bioinformatics/btr655 PMC3268234

[17] Katoh K, Misawa K, Kuma K, Miyata T (2002) MAFFT: A novel method for rapid multiple sequence alignment based on fast Fourier transform. Nucleic Acids Res 30: 3059–66. 12136088 10.1093/nar/gkf436 PMC135756

[18] Huson DH, Bryant D (2006) Application of phylogenetic networks in evolutionary studies. Mol Biol Evol 23: 254–267. 16221896 10.1093/molbev/msj030

[19] Stamatakis A (2011) Phylogenetic analysis of protein sequence data using the Randomized Axelerated Maximum Likelihood (RAXML) Program. Curr Protoc Mol Biol. Chapter 19: Unit 19.11. 10.1002/0471142727.mb1911s96 21987055

[20] Huson DH, Scornavacca C (2012) Dendroscope 3: an interactive tool for rooted phylogenetic trees and networks. Syst Biol 61: 1061–1067. 22780991 10.1093/sysbio/sys062

[21] Chatterjee SN, Chaudhuri K (2004) Lipopolysaccharides of *Vibrio cholerae*: II. Genetics of biosynthesis. Biochim Biophys Acta 1690: 93–109. 15469898 10.1016/j.bbadis.2004.06.006

[22] Heidelberg JF, Eisen JA, Nelson WC, Clayton RA, Gwinn ML, et al (2000) DNA sequence of both chromosomes of the cholera pathogen *Vibrio cholerae* . Nature 406: 477–483. 10952301 10.1038/35020000 PMC8288016

[23] Reimer AR, Van Domselaar G, Stroika S, Walker M, Kent H, et al (2011) Comparative genomics of *Vibrio cholerae* from Haiti, Asia, and Africa. Emerg Infect Dis 17: 2113–2121. 22099115 10.3201/eid1711.110794 PMC3310578

[24] Feng L, Reeves PR, Lan R, Ren Y, Gao C, et al (2008) A recalibrated molecular clock and independent origins for the cholera pandemic clones. PLoS One 3: e4053. 19115014 10.1371/journal.pone.0004053 PMC2605724

[25] Chun J, Grim CJ, Hasan NA, Lee JH, Choi SY, et al (2009) Comparative genomics reveals mechanism for short-term and long-term clonal transitions in pandemic *Vibrio cholerae* . Proc Natl Acad Sci U S A 106: 15442–15447. 19720995 10.1073/pnas.0907787106 PMC2741270

[26] Huber KE, Waldor MK (2002) Filamentous phage integration requires the host recombinases XerC and XerD. Nature 417: 656–659. 12050668 10.1038/nature00782

[27] Val ME, Bouvier M, Campos J, Sherratt D, Cornet F, et al (2005) The single-stranded genome of phage CTX is the form used for integration into the genome of *Vibrio cholerae* . Mol Cell 19: 559–566. 16109379 10.1016/j.molcel.2005.07.002

[28] McLeod SM, Waldor MK (2004) Characterization of XerC- and XerD-dependent CTX phage integration in *Vibrio cholerae* . Mol Microbiol 54: 935–947. 15522078 10.1111/j.1365-2958.2004.04309.x

[29] Waldor MK, Mekalanos JJ (1996) Lysogenic conversion by a filamentous phage encoding cholera toxin. Science 272: 1910–1914. 8658163 10.1126/science.272.5270.1910

[30] Dittmer JB, Withey JH (2012) Identification and characterization of the functional toxboxes in the *Vibrio cholerae* cholera toxin promoter. J Bacteriol 194: 5255–5263. 22821976 10.1128/JB.00952-12 PMC3457234

[31] Mekalanos JJ, Swartz DJ, Pearson GD, Harford N, Groyne F, et al (1983) Cholera toxin genes: nucleotide sequence, deletion analysis and vaccine development. Nature 306: 551–557. 6646234 10.1038/306551a0

[32] O’Shea YA, Reen FJ, Quirke AM, Boyd EF (2004) Evolutionary genetic analysis of the emergence of epidemic *Vibrio cholerae* isolates on the basis of comparative nucleotide sequence analysis and multilocus virulence gene profiles. J Clin Microbiol 42: 4657–4671. 15472325 10.1128/JCM.42.10.4657-4671.2004 PMC522369

[33] Karaolis DK, Johnson JA, Bailey CC, Boedeker EC, Kaper JB, et al (1998) A *Vibrio cholerae* pathogenicity island associated with epidemic and pandemic strains. Proc Natl Acad Sci U S A 95: 3134–3139. 9501228 10.1073/pnas.95.6.3134 PMC19707

[34] Jermyn WS, Boyd EF (2002) Characterization of a novel *Vibrio* pathogenicity island (VPI-2) encoding neuraminidase (*nanH*) among toxigenic *Vibrio cholerae* isolates. Microbiology 148: 3681–3693. 12427958 10.1099/00221287-148-11-3681

[35] Grim CJ, Choi J, Chun J, Jeon YS, Taviani E, et al (2010) Occurrence of the *Vibrio cholerae* seventh pandemic VSP-I island and a new variant. OMICS 14: 1–7. 20141327 10.1089/omi.2009.0087

[36] Dziejman M, Balon E, Boyd D, Fraser CM, Heidelberg JF, et al (2002) Comparative genomic analysis of *Vibrio cholerae*: genes that correlate with cholera endemic and pandemic disease. Proc Natl Acad Sci U S A 99: 1556–1561. 11818571 10.1073/pnas.042667999 PMC122229

[37] Tamura K, Peterson D, Peterson N, Stecher G, Nei M, et al (2011) MEGA5: molecular evolutionary genetics analysis using maximum likelihood, evolutionary distance, and maximum parsimony methods. Mol Biol Evol 28: 2731–2739. 21546353 10.1093/molbev/msr121 PMC3203626

[38] Nandi B, Nandy RK, Mukhopadhyay S, Nair GB, Shimada T, et al (2000) Rapid method for species-specific identification of *Vibrio cholerae* using primers targeted to the gene of outer membrane protein OmpW. J Clin Microbiol 38: 4145–4151. 11060082 10.1128/jcm.38.11.4145-4151.2000 PMC87555

[39] Nandi B, Nandy RK, Sarkar A, Ghose AC (2005) Structural features, properties and regulation of the outer-membrane protein W (OmpW) of *Vibrio cholerae* . Microbiology 151: 2975–2986. 16151208 10.1099/mic.0.27995-0

[40] Hunter S, Jones P, Mitchell A, Apweiler R, Attwood TK, et al (2012) InterPro in 2011: new developments in the family and domain prediction database. Nucleic Acids Res 40: D306–D312. 22096229 10.1093/nar/gkr948 PMC3245097

[41] Garcia-Pino A, Christensen-Dalsgaard M, Wyns L, Yarmolinsky M, Magnuson RD, et al (2008) Doc of prophage P1 is inhibited by its antitoxin partner Phd through fold complementation. J Biol Chem 283: 30821–20827. 18757857 10.1074/jbc.M805654200 PMC2576525

[42] Fonseca EL, Dos Santos Freitas F, Vieira VV, Vicente AC (2008) New qnr gene cassettes associated with superintegron repeats in *Vibrio cholerae* O1. Emerg Infect Dis 14: 1129–1131. 18598639 10.3201/eid1407.080132 PMC2600354

[43] Xia R, Guo X, Zhang Y, Xu H (2010) *qnrVC*-like gene located in a novel complex class 1 integron harboring the ISCR1 element in an *Aeromonas punctata* strain from an aquatic environment in Shandong Province, China. Antimicrob Agents Chemother 54: 3471–3474. 20516288 10.1128/AAC.01668-09 PMC2916331

[44] Rowe-Magnus DA, Guerout AM, Mazel D (2002) Bacterial resistance evolution by recruitment of super-integron gene cassettes. Mol Microbiol 43: 1657–1669. 11952913 10.1046/j.1365-2958.2002.02861.x

[45] Hall TA (1999) BioEdit: a user-friendly biological sequence alignment editor and analysis program for Windows 95/98/NT. Nucl. Acids. Symp. Ser. 41: 95–98.

[46] Belfaiza J, Martel A, Margarita D, Saint Girons I (1998) Direct sulfhydrylation for methionine biosynthesis in *Leptospira meyeri* . J Bacteriol 180: 250–255. 9440513 10.1128/jb.180.2.250-255.1998 PMC106879

[47] Sastry MS, Korotkov K, Brodsky Y, Baneyx F (2002) Hsp31, the *Escherichia coli yedU* gene product, is a molecular chaperone whose activity is inhibited by ATP at high temperatures. J Biol Chem 277: 46026–46034. 12235139 10.1074/jbc.M205800200

[48] Rasouly A, Shenhar Y, Ron EZ (2007) Thermoregulation of *Escherichia coli hchA* transcript stability. J Bacteriol 189: 5779–5781. 17526696 10.1128/JB.00453-07 PMC1951820

[49] Mujacic M, Baneyx F (2007) Chaperone Hsp31 contributes to acid resistance in stationary-phase *Escherichia coli* . Appl Environ Microbiol 73: 1014–1018. 17158627 10.1128/AEM.02429-06 PMC1800746

[50] Merrell DS, Camilli A (1999) The *cadA* gene of *Vibrio cholerae* is induced during infection and plays a role in acid tolerance. Mol Microbiol 34: 836–849. 10564522 10.1046/j.1365-2958.1999.01650.x

