# Supplementary figures and images for: Comparative Genomic Characterization of a Thailand–Myanmar Isolate, MS6, of Vibrio cholerae O1 El Tor, Which Is Phylogenetically Related to a “US Gulf Coast” Clone
Source: PLoS One. 2014 Jun 2;9(6):e98120. doi: 10.1371/journal.pone.0098120 (PMC4045137; doi:10.1371/journal.pone.0098120)

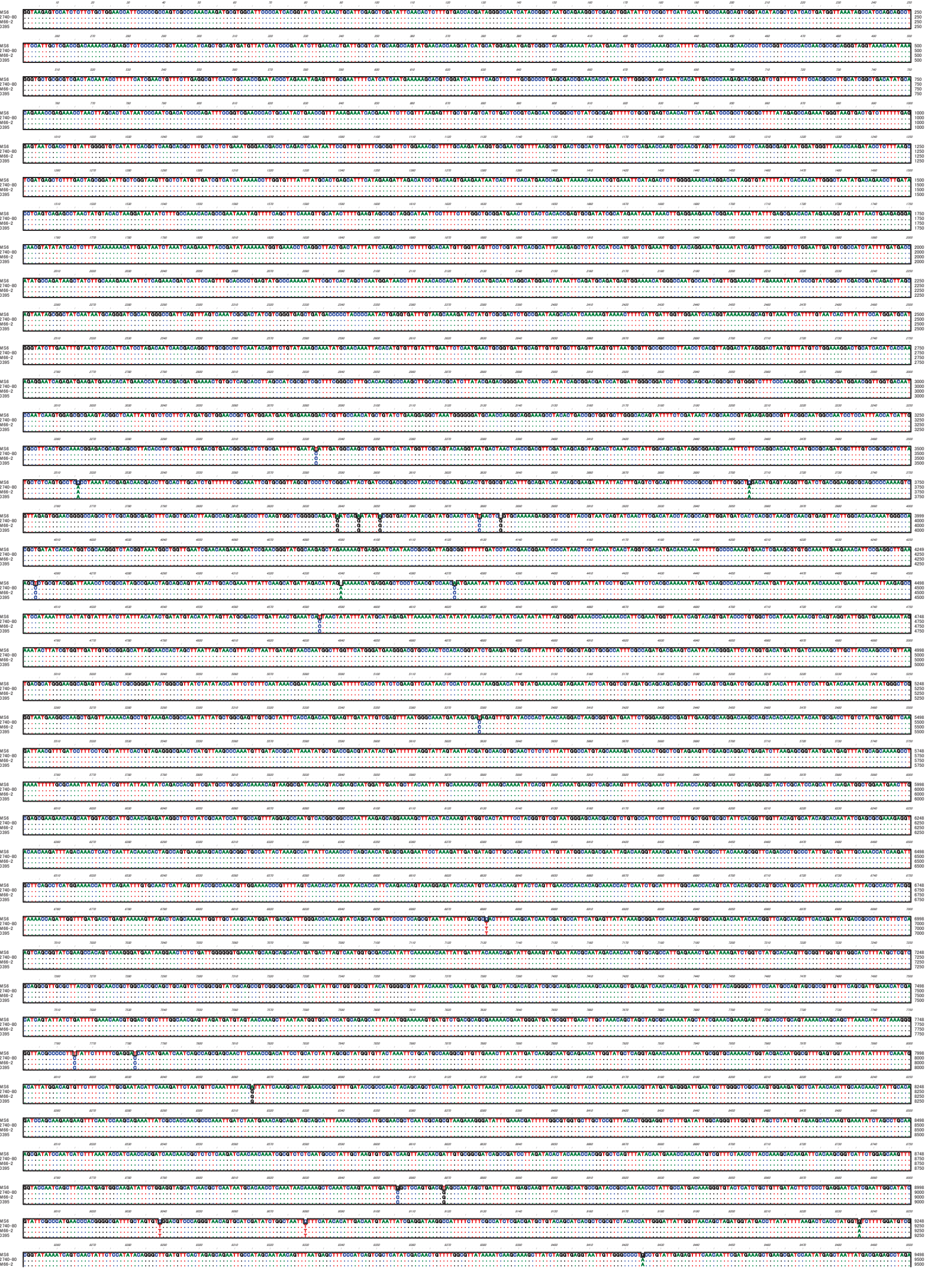

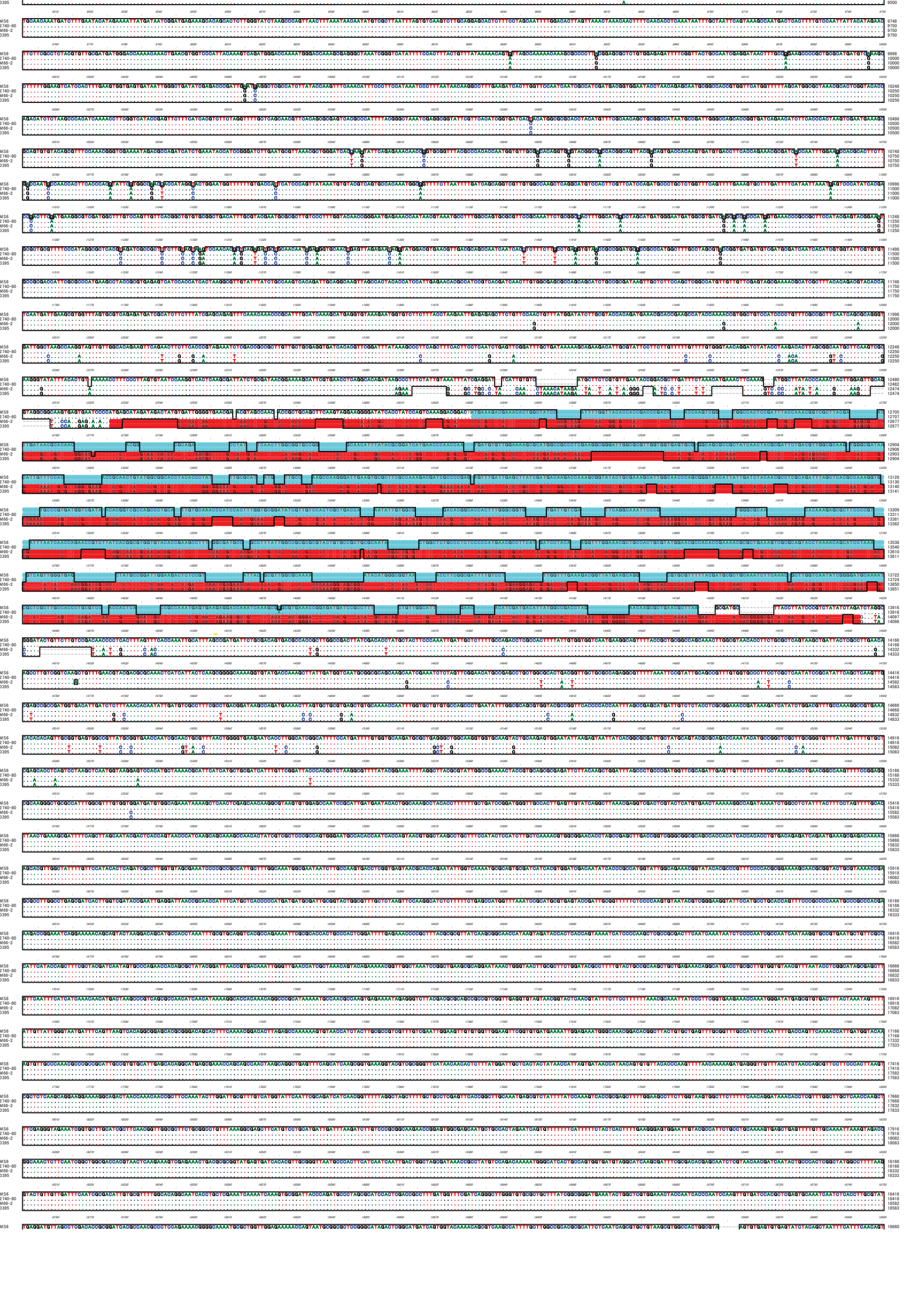

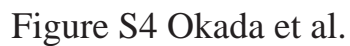

Supplement: Figure S6 — Evidence for the substitution of hchA/luxR or metY in highly conserved regions. The nucleotide sequences of an approximately 22-kb region containing hchA/luxR or metY in MS6, 2740-80 (U. S. Gulf Coast), M66-2 (pre-seventh pandemic), and O395 (classical) strains were aligned using BioEdit version 7.1.3.0 [45]. The location of metY and hchA/luxR are highlighted in blue and red, respectively. Identical nucleotides are indicated by dots. The distribution of sequence differences (mismatches and gaps (−) is most frequent near hchA/luxR and metY, whereas the regions upstream and downstream of these genes are highly conserved. (PDF) [file pone.0098120.s006.pdf]
